# Editorial: High-altitude neuroscience: innovative pathways from adaptive mechanisms to health management

**DOI:** 10.3389/fnins.2026.1968224

**Published:** 2026-09-22

**Authors:** Niannian Wang, Hao Li, Tao Zhang, Martin Burtscher, Hailin Ma

**Affiliations:** 1 Xizang Key Laboratory of Plateau Hypoxic Environment and Life Health, Xizang University, Lhasa, China; 2 Mental Health Education Center and the School of Science, Xihua University, Chengdu, China; 3 Department of Sport Science, University of Innsbruck, Innsbruck, Austria

**Keywords:** EEG, health management, high altitude, hormesis, hypobaric hypoxia, neural adaptation, neuroplasticity, sleep

High-altitude (HA) environments, conventionally defined as elevations above 2,500 m, provide a distinctive natural setting for investigating how the human brain responds to sustained environmental stress. Reduced barometric pressure and oxygen availability coexist with cold, aridity, ultraviolet radiation, and large diurnal temperature variation, placing substantial demands on physiological homeostasis (Chroboczek et al., 2021; Virues-Ortega et al., 2004). The brain is particularly sensitive because of its high and continuous energy requirements (Raichle, 2010; Raichle and Gusnard, 2002; Yan, 2014). Yet, HA exposure cannot be understood as a simple progression from hypoxia to dysfunction. Depending on dose, duration, developmental stage, genetic background, health status, and prior acclimatization, neural responses may encompass stress, compensation, adaptation, resilience, vulnerability, and, under selected conditions, therapeutic plasticity.

The Research Topic “*High-altitude neuroscience: innovative pathways from adaptive mechanisms to health management*” brings together 13 peer-reviewed contributions: nine Original Research articles, two Review articles, one Brief Research Report, and one Opinion article. Collectively, they span molecular mechanisms, neurophysiology, neuroimaging, cognition, development, sleep, psychiatry, occupational health, and intervention. Rather than functioning as a catalog of studies, the collection points toward a multilevel view in which cerebral outcomes emerge from interactions among oxygen availability, systemic physiology, neural reorganization, individual susceptibility, and exposure history. The overall framework of the Editorial is shown in Figure 1.

**Figure 1:**
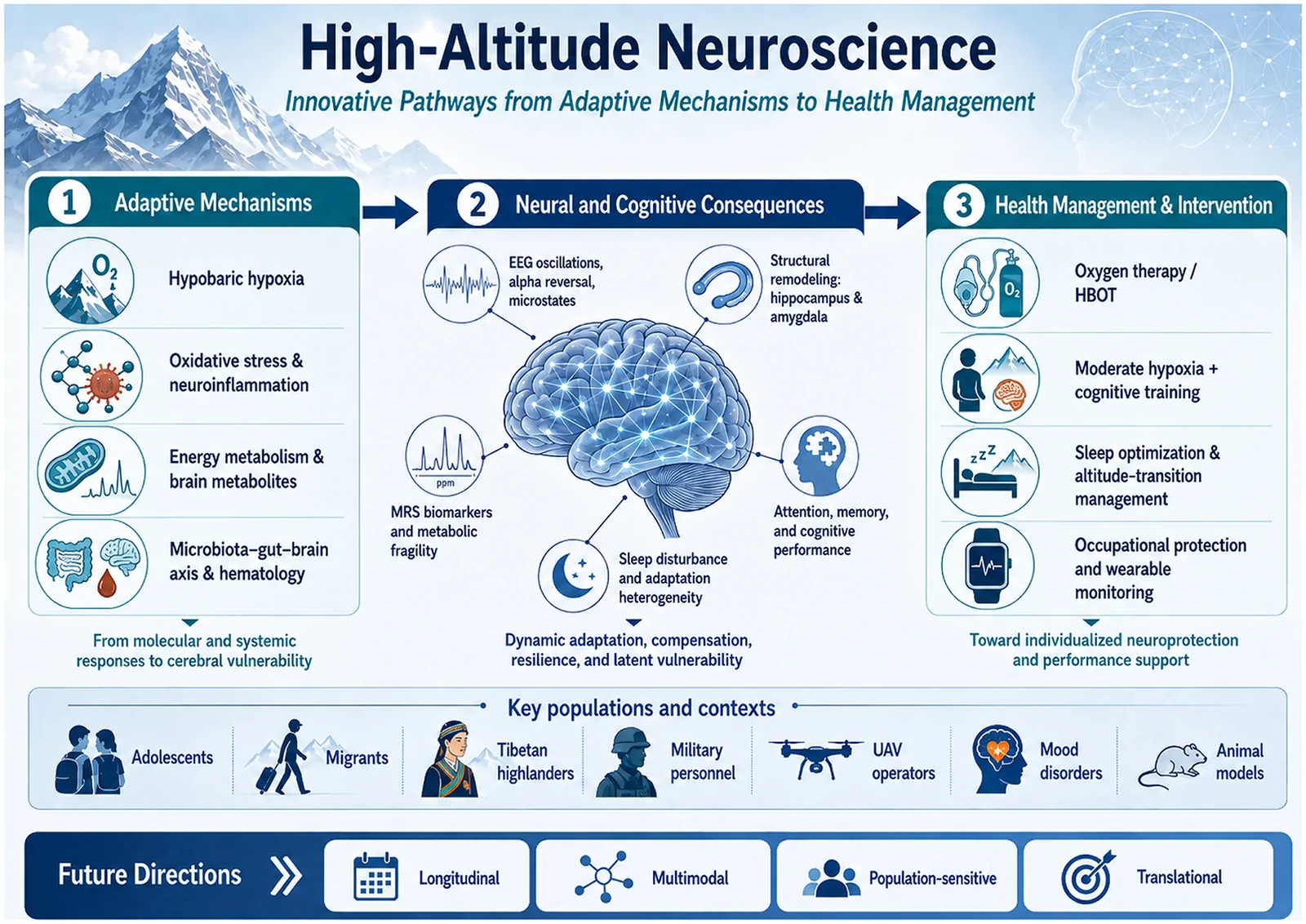
Conceptual framework of high-altitude neuroscience: adaptive mechanisms, neural consequences, and health management. The figure illustrates multilevel effects of high-altitude exposure, from hypoxia-related molecular and physiological responses to neural reorganization, cognition, sleep, and health-management strategies. The “Key populations and contexts” panel summarizes populations and exposure contexts represented across the contributions to this Research Topic and is not intended as an exhaustive classification of populations affected by high altitude. In the “Health Management and Intervention” panel, oxygen-based therapeutic interventions (including hyperbaric oxygen therapy, HBOT) refer to clinical interventions examined within the collection; HBOT is not a feature of natural high-altitude hypobaric exposure.

## From molecular mechanisms to neural vulnerability

Several contributions clarify why apparently successful acclimatization may coexist with biological cost. Gong et al. synthesize mechanisms implicated in HA-related cognitive change, including disturbed energy metabolism, oxidative stress, inflammation, autophagy, gut microbiota alterations, and genetic susceptibility, while emphasizing the need for improved preclinical and human experimental models. Xu et al. extend this systems perspective through the microbiota-gut-brain axis, highlighting intestinal barrier dysfunction, microbial metabolites, inflammatory signaling, autonomic pathways, and neuroendocrine regulation as candidate links between HA exposure and brain health.

Experimental work reinforces the importance of reserve and vulnerability. Hu et al. show in a mouse model that severe simulated HA hypoxia induces transcriptomic disturbance, oxidative stress, glial activation, and neuroinflammation, whereas normobaric and hyperbaric oxygen interventions partially restore molecular homeostasis. Park et al., using longitudinal ^1^H-MRS, show that chronic hypobaric hypoxia alters baseline neurochemistry and magnifies metabolic disturbance after mild traumatic brain injury. Together, these findings suggest that preserved outward functioning does not necessarily imply preserved biological reserve.

## Dynamic neural adaptation, cognition, and structure

The Research Topic also demonstrates that HA adaptation is temporally dynamic. Hutcheon revisits the Berger effect and argues that altered eyes-open/eyes-closed alpha relationships under hypoxia may provide a useful model for probing the neurophysiology of alpha oscillations. In longitudinal resting EEG, Liu Q. et al. report a shift from relatively greater regional complexity during earlier exposure toward reduced local complexity but stronger interregional coupling after longer exposure, suggesting reorganization toward more coordinated processing. Liu Z. et al. further link systemic physiology to neural dynamics, identifying an indirect statistical pathway between body mass index, estimated blood viscosity, and EEG microstate B occurrence.

Population and developmental context are equally important. Chen et al. show that indigenous highland adolescents living at different elevations differ in memory performance and stage-specific electrophysiological responses, underscoring the need to distinguish developmental adaptation from adult acclimatization. Zuo et al. report selective hippocampal and amygdalar subregional volume reductions in long-term migrants despite relatively preserved broader behavioral functioning. Such brain-behavior dissociations should not automatically be interpreted as either successful compensation or pathology; longitudinal evidence is needed to determine whether they reflect adaptive reorganization, physiological cost, or reduced resilience to subsequent stress.

## Hypoxia as a double-edged stimulus: a hormetic perspective

A central theme of the collection is that hypoxia is not uniformly deleterious. Its effects depend on intensity, duration, intermittency, context, and adaptive state. Jensen et al. examined repeated moderate normobaric hypoxia combined with cognitive training in individuals with remitted mood disorders. Although previous trial findings indicated improvement in executive function, the imaging analysis did not detect corresponding changes in prefrontal or hippocampal diffusion-derived microstructure. In contrast, Hu et al. demonstrate that oxygen supplementation can attenuate molecular and inflammatory consequences of severe hypoxic stress.

This non-linear pattern is consistent with a hormetic framework: controlled, sublethal hypoxic stress may engage adaptive or preconditioning mechanisms, whereas severe or prolonged hypoxia may overwhelm compensatory capacity and promote injury (Mattson and Leak, 2024; Rybnikova and Samoilov, 2015). Importantly, hormesis should be treated as a testable dose- and context-dependent framework rather than as evidence that hypoxia is inherently beneficial. Translational studies must therefore define what works, for whom, at what dose, and during which phase of exposure.

## Sleep, occupational health, and precision management

The sleep and occupational studies shift the field toward real-world health management. Lin et al. show that sleep disturbance and its metabolic correlates differ among lowlanders, HA migrants, and indigenous Tibetan highlanders, illustrating how environmental acclimatization and multigenerational adaptation may produce distinct risk profiles. Yang et al. further show in military personnel that sleep quality is related not only to cumulative HA exposure but also to oxygen supplementation and transitions between high- and low-altitude environments. Shu et al. broaden the perspective to unmanned aerial vehicle operators, where neurological, musculoskeletal, respiratory, and psychological symptoms intersect with workload and scheduling.

These studies argue against defining exposure solely by altitude and duration. Contemporary HA populations often experience repeated ascent and descent, variable oxygen use, sleep disruption, workload, and occupational stress. Precision health management will therefore require integration of exposure history, physiological monitoring, sleep, cognition, and organizational context rather than a single biomarker or universal intervention.

## Toward the next generation of high-altitude neuroscience

Several priorities follow from this Research Topic. First, longitudinal within-person designs are essential for separating acute responses, acclimatization, chronic adaptation, cumulative biological cost, and de-acclimatization. Second, exposure characterization should be standardized to include barometric pressure, inspired oxygen concentration when applicable, ascent profile, previous HA experience, oxygen supplementation, sleep, workload, and recent altitude transitions.

Third, multilevel integration should explicitly include genetic and epigenetic determinants alongside molecular signaling, inflammation, metabolism, hematology, cerebral hemodynamics, electrophysiology, structural and functional imaging, cognition, emotion, and sleep. Integrating these domains within the same participants will be crucial for explaining individual differences in adaptive capacity.

Fourth, population heterogeneity should be treated as a scientific question rather than a nuisance variable. Relevant examples include, but are not limited to, migrants, indigenous highlanders, children and adolescents, older adults, military and occupational groups, patients with psychiatric or neurological conditions, and individuals with metabolic risk. Sex-related differences also remain insufficiently studied. Finally, the field must distinguish adaptation, compensation, resilience, and vulnerability more precisely and test interventions according to exposure phase and individual adaptive state.

## Conclusion

This Research Topic illustrates a transition from a predominantly deficit-oriented view of HA neuroscience toward a broader science of environmental brain adaptation. Hypoxia can disturb metabolism, inflammation, brain structure, cognition, and sleep, yet neural systems also reorganize across exposure, and carefully controlled hypoxic or oxygen-based interventions may engage adaptive or restorative pathways. Viewed through a hormetic lens, the central question is not whether hypoxia is simply harmful or beneficial, but where the boundary lies between adaptive challenge and biological overload. Resolving that boundary through longitudinal, multimodal, genetically and epigenetically informed, and population-sensitive research will be essential for translating high-altitude neuroscience into individualized neuroprotection and health management.

We sincerely thank all authors for their valuable contributions to this Research Topic and all reviewers for their time, expertise, and constructive evaluations. We also thank the editorial and publishing teams for their support throughout the development of this Research Topic.
